# Postpartum care needs assessment: women’s understanding of postpartum care, practices, barriers, and educational needs

**DOI:** 10.1186/s12884-023-05813-0

**Published:** 2023-07-07

**Authors:** Yenupini Joyce Adams, Michelle Louise Miller, John Stephen Agbenyo, Ethel Emefa Ehla, Grace Anne Clinton

**Affiliations:** 1 Eck Institute for Global Health, University of Notre Dame, Notre Dame, IN USA; 2 Indiana University School of Medicine, Indianapolis, IN USA; 3 Savana Signatures, Tamale, Ghana; 4 University of Notre Dame, Notre Dame, IN USA

**Keywords:** Postpartum care, Postnatal care, Midwives, Maternal Health, Maternal Mortality, Postpartum Depression, Ghana, Sub-Saharan Africa

## Abstract

**Background:**

Complications in the postpartum period pose substantial risks to women and can result in significant maternal morbidity and mortality. However, there is much less attention on postpartum care compared to pregnancy and childbirth. The goal of this study was to gather information on women’s knowledge of postpartum care and complications, recovery practices after childbirth, perceived barriers to receiving care during the postpartum period, and educational needs in four health centers. The findings can inform the development of appropriate curriculum and interventions for postnatal care education in similar settings.

**Methods:**

A descriptive qualitative study design was employed. Eight focus group discussions were conducted among 54 postpartum women who delivered in four health centers in Sagnarigu District in Tamale, Ghana. Audio recordings of focus group data were transcribed and translated, and thematic analysis was conducted.

**Results:**

There were six main themes that emerged from the focus group discussions: 1) baby focused postpartum care; 2) postpartum practices; 3) inadequate knowledge ofpostpartum danger signs; 4) barriers to accessing postpartum care 5) experiences of poor mental health; and 6) need for postpartum education.

**Conclusions:**

Postpartum care for women in this study was primarily perceived as care of the baby post-delivery and missing key information on physical and mental health care for the mother. This can result in poor adjustment postpartum and critically, a lack of knowledge on danger signs for common causes of morbidity and mortality in the postpartum period. Future research needs to understand how to communicate important information on postpartum mental and physical health to better protect mothers in the region.

## Background

While the number of maternal deaths has been decreasing over the past twenty years, specific regions of the world are still greatly affected by maternal mortality. The Sub-Saharan African (SSA) region has the highest MMR at 542 deaths per 100,000 live births [1]. Nations in the SSA region like Ghana continue to battle high maternal death rates, higher than the global average, with a MMR point estimate of 308 deaths per 100,000 live births [1]. The postpartum/postnatal period is pivotal in supporting the long term physical and mental health of mothers and their children [2]. More than a third of maternal deaths are the result of postpartum complications [1]. Quality maternal health care during the postpartum period is essential in decreasing maternal death rates throughout the world, especially in nations such as Ghana with relatively high maternal mortality ratios.

Maternity care has traditionally focused on encouraging women to seek obstetric care at a healthcare facility during pregnancy, labor and delivery [3, 4]. These care practices are important, as receiving care during childbirth in a healthcare facility lowers the risk of complications like excessive bleeding, perinatal asphyxia, and fetal distress [5]. Giving birth at a healthcare facility also provides time for the mother and baby to be examined before discharge for signs of sepsis or hemorrhage, the leading causes of maternal mortality [6]. While these recommendations have been successful in improving outcomes, they still do not address all the medical issues of women during the postpartum period [7]. There is still a large portion of women who die from birth-related complications later in their homes. Recent studies indicate that the length of most postpartum hospital stays may be too short for effective treatment of most life-threatening complications [8].

There are various barriers to receiving postpartum care in Ghana. Of note, most women in Ghana have access to national health insurance which covers care before and after pregnancy [9]. More than three quarters of women (79%) between the ages of 15–49 have some health insurance and about 8 in 10 women have insurance that covers antenatal care, childbirth, and postnatal care [9]. However, current research shows that early postpartum care is underutilized by women in SSA countries. In Ghana, only 4% of women receive postpartum care 3–41 days after delivery.15, Some influencing factors include:younger age, lack of women’s autonomy, lower level of education, negative provider attitude, and lack of information on danger signs [10–12]. Further, barriers such as lack of available mental health resources, lack of education for postpartum women on how to access care, and lack of existing relationships between patients and healthcare facilities also contribute to the underutilization of postpartum care [13]. Yet, a recent study found that women who demonstrated an understanding of postpartum dangers signs were the most likely to use postpartum care services [14]. In the Northern region of Ghana where this study was conducted, about 71% of women seek postpartum care within two days after giving birth, compared to 91% in the Upper West and Greater Accra regions [13]. Understanding how to increase utilization of postpartum care services has the potential to reduce preventable deaths among postpartum women.

Consideration of mental health in addition to physical health during the postpartum period is crucial. There is a high prevalence of antepartum depression and anxiety in SSA, which has the potential to affect maternal health after pregnancy [15]. Mental health issues like depression and anxiety, can be debilitating for everyday functioning within the family and community. Approximately 26.6% of Ghanaian women experience antepartum depression. Alarmingly, there is a lack of mental health resources throughout the nation. A s of 2016, there were only 16 registered psychiatrists (around 1 per 1.5 million population) [16]. It is unlikely that the current mental health resources can address all of the needs of postpartum women.

There is a gap in the research to clarify the relationship between women and their experiences with mental health after birth. While there are broader studies looking at mental health in SSA countries, there are no subjective qualitative studies focused on postnatal maternal health in SSA countries, such as Ghana.

Postpartum education can help to reduce maternal mortality and morbidity. Postpartum visits are a critical time to provide information on reducing risk of postpartum complications that can lead to serious injury or death. The World Health Organization (WHO) sets clear guidelines that encourage postpartum assessments at twenty-four hours post-delivery, at day three, at two weeks, and at six weeks [17]. A 2015 study examined women who received postpartum checkups in Brong Ahafo, Ghana. Even after these checkups, women still lacked an understanding of basic postpartum care. The authors also found that in hospital settings, many women were not provided with standardized postpartum discharge information [18]. A different study in Accra, Ghana, highlighted the shortcomings of current education, as 99% of mothers received education but still none could recall more than 4 maternal danger signs [19]. Additionally, only 33% of providers knew the PNC schedule and there was no consistent format for standardized PNC [19]. While there are some PNC practices in place in terms of education, there are significant gaps in postpartum teaching to properly lower maternal mortality in Ghana.

There are significant barriers to accessing adequate postpartum care, especially in the domains of existing postpartum practices, mental health, and education during postpartum visits. The purpose of this study was to conduct a postpartum care needs assessment among postpartum women who delivered at health care centers in Tamale, Ghana. Specifically, the objective was to understand the experiences of women who delivered in healthcare centers in Tamale in relation to their understanding of postpartum care, personal health practices, perceived barriers, and educational needs. The results of the study will be used to develop a more comprehensive postpartum education program to improve postpartum outcomes among women in the setting.

## Methods

### Aim, design, and setting

The primary aim was to understand the experiences of women who delivered in healthcare centers in Tamale in relation to their understanding of postpartum care, personal health practices, perceived barriers, and educational needs. A descriptive qualitative study design was employed. Data were collected via focus group discussions among a sample of postpartum women from four health centers in Sagnarigu municipality, Tamale, Ghana, where the research team will implement a planned postpartum care intervention. These health centers included Kanvilli, Choggu, Kalpohin, and Bagabaga health centers. We conducted focus group discussions because women are more likely to talk in a group setting when they know the discussion is not targeted at them personally, and it will allow women to learn from each other. Focus groups included a multiple-category design with two categories: women less than 6 months postpartum and women 6 months to 1 year postpartum, considering that women in the later postpartum period may have differing experiences from women in the early postpartum period.

### Participant characteristics

Purposive sampling was used to select participants for the focus groups within the health centers. This sampling technique is considered appropriate for the “selection of small samples from a limited geographic area or restricted population definition” (Lavrakas, 2008, p. 2). Participants included postpartum women 18 years and older, who delivered a baby within a year and were able to speak and understand Dagbani or English. Women were purposively sampled to represent a mix of parity and the number of weeks since birth.

### Recruitment process

The study was reviewed and approved by the University of Notre Dame Institutional Review Board. Permission to conduct the focus groups was also obtained from the Sagnarigu District Health Directorate. All study procedures, including the recruitment and informed consent process, were performed in accordance with the approved protocol by the University of Notre Dame Institutional Review Board.

The study team had previously worked with the participating health facilities, and had a good working relationship with them. The health facility heads/in-charge were engaged after permission was obtained from the District Health Office. Once these engagements were made and permission received to recruit participants during postnatal visit days, recruitment and data collection began. Women were recruited by the study team for the focus group discussions when they came to the health center for their postnatal care visit. Each of the health facilities have specific postnatal care days when women return to be seen.

The research team approached women individually and explained the study to them. Typically, women come to the facility on postnatal care day, and wait for their turn to be seen by the midwives. Women were approached during this time. Women who expressed interest in participating were assessed for eligibility and then invited to participate in the discussion. Discussions were held after all eligible women were done with their postnatal care that day.

### Data collection

A member of the research team individually explained the consent statement and answered any questions, and verbal consent was obtained from each participant before the focus group discussion. A demographic form of participant characteristics, without identifiers, was collected from each participant. A total of 8 focus groups were conducted. Two focus groups were conducted at each health center, consisting of a below 6 months postpartum group, and an above 6 months postpartum group. Each focus group had between 6–8 women in attendance. Focus groups were conducted in English or Dagbani, based on language preferences of participants (7 in Dagbani and 1 in English).

Focus groups were conducted by two facilitators (female and male) who were fluent in both English and Dagbani and had prior experience conducting sessions with women in the setting. Both facilitators were program managers with the in-country partner organization. Since the organization has been working with women in the setting for years and are well known by the community, participants were vocal during the sessions. With the participants' consent, focus group discussions were audio-recorded. Field notes were taken at each group discussion in addition to the audio recordings. Notetakers were two expert translators the team hired, who were also responsible for transcribing and translating the data. All focus group discussions were conducted in June, 2021.

### Focus group guide

A semi-structured focus group discussion guide was used by the facilitators to guide the group discussions. The guide was developed by the researchers and in-country partners. The guide was developed using knowledge from the literature and from the PI and in-country partners’ extensive experience in the setting. The same guide was used for all focus group discussions. Participants during the sessions discussed their experiences of postpartum care, barriers to postpartum care for the mother, health behaviors practiced during the postpartum period, experiences with physical recovery and mental health, knowledge of danger signs, and educational needs postpartum.

### Data analysis

English audio-recorded discussions were transcribed verbatim and reviewed by the research team. Recordings in Dagbani were transcribed verbatim and translated into English by two expert translators who were also note-takers during the discussions and reviewed by the research team. Thematic analysis was conducted following the thematic analysis steps outlined by Braun & Clark (2006). First, the transcripts were reviewed to familiarize ourselves with the data, after which initial codes were generated by first, second and fifth authors. After codes were generated, the first and second authors searched for themes and then reviewed, defined, and named the themes. Finally, the report was generated, and all team members reviewed the report.

## Results

Participant characteristics were extracted from the demographic forms collected and are displayed in Table 1. There were 54 participants (age range, 19 to 44), with the mean age approximately 28 years (SD = 5.15). Approximately 88% (*n* = 46) of participants were married. About 87% (*n* = 45) were unemployed or artisan and about 35% (*n* = 18) had no education. Most participants (89%, *n* = 48) delivered vaginally, and most had more than one child (69%, *n* = 37).

**Table 1:** Sociodemographic and Obstetric Information of Participants in Focus Groups, N = 54

*Characteristic*	*Number (n)*	*Percent (%)*
***Health Facility***		
*Kanvilli*	13	24.1
*Choggu*	13	24.1
*Kalpohin*	14	25.9
*Bagabaga*	14	25.9
***Postpartum Timeline***		
*Less than 6 months postpartum*	27	50
*More than 6 months postpartum*	27	50
***Focus Group Language***		
*English*	7	13.0
*Dagbani*	47	87.0
***Participant Age (years)***		
*Mean (SD)*	28.15 (5.15)	
*18 to 24*	13	24.1
*25 to 34*	34	63.0
*35 to 44*	7	13.0
***Employment Status***		
*Employed*	7	13.5
*Unemployed/artisan*	45	86.5
***Marital Status***		
*Single*	1	1.9
*Married*	46	88.5
*Cohabitating*	4	7.7
*Divorced*	1	1.9
***Education***		
*No Education*	18	34.6
*Primary*	13	25
*Secondary*	16	30.8
*Tertiary*	5	9.6
***First Delivery***		
*Yes*	17	31.5
*No*	37	68.5
***Number of Deliveries***		
*Mean (SD)*	2.46 (1.48)	
*1*	17	31.5
*2 to 4*	31	57.4
*5 to 8* +	6	11.1
***Number of Living Children***		
*Mean (SD)*	2.39 (1.39)	
*1*	17	31.5
*2 to 4*	32	59.3
*5 to 8* +	5	9.3
***Type of Delivery for this Baby***		
*Vaginal*	48	88.9
*Cesarean Section*	6	11.1

Six themes emerged from the analysis and included: 1) baby-focused postpartum care; 2) postpartum practices; 3) inadequate knowledge of postpartum danger signs; 4) barriers in accessing postpartum care; 5) experiences of poor mental health; and 6) need for postpartum education. Themes with selected illustrative quotes are described below. Additional illustrative quotes are presented in Table 2.

**Table 2:** Themes with Selected Illustrative Quotes

*Theme*	*Selected Illustrative Quotes*
**Baby-Focused Postpartum Care**	*“What postpartum care entails is that you may want to take good care of the baby and you are not well to do because most of us are unemployed, so you can’t do it. We are supposed to take good care of them. On the part of breast feeding, we should keep our breasts neat.” (Kalpohin* < *6mo)*
	*“If you can afford, you take the child to school and makaranta (Islamic school).” (Kanvili, 6mo* +*)*
	*“After delivery, like every month they do us to come for weighing. They check on my child’s weight as to whether it is reducing or it is increasing. If it reduces they will teach you on what and what to do to let him gain weight and then they also help the child to grow. They give like the 6 killer diseases. They give injections like every month or every 3 months just to prevent the child from getting these diseases or diseases that are coming. So that’s what I understand.” (Choggu, 6mo* +*)*
	*“They tell us how to feed the baby to be strong but not we the mothers. We receive education only about how to care for the baby.” (Bagabaga 6mo* +*)*
	*“They educate us on the importance of family planning. If you do family planning, you can take proper care of you children if they are few.” (Kanvili, 6mo* +*)*
	*“They teach us to practice personal hygiene that will prevent the breast milk from contamination so that the child can feed on it. Wash your hands with soap and water after visiting the toilet and wipe your hands with a clean wiper or handkerchief before you breastfeed the child.” (Kalpohin* < *6mo)*
	*“When I was pregnant that they were taking my vitals but after delivery they have not done such a thing.” (Kalpohin* < *6mo)*
	*“Yes, it is only the children that they take care of. The nurses don't take care of us at the facility. When you complain about any condition to them, they will tell you that they heard you and that is all.” (Kalpohin* < *6mo)*
	*“When you complain about yourself not being well they will tell you to go to OPD.” (Choggu, 6mo* +*)*
**Postpartum Practices**	*“In the culture of the Dagombas, some women don’t give birth at the hospital. So, when a woman gets a cut, they call it ŋmaɣa. If it happens to a woman’, they can use local soap commonly known as ‘awabila’ to wash it.” (Bagabaga 6mo* +*)*
	*“Wash your hand with soap and water to keep it hygienic anytime you are to pick the child.” (Kalpohin* < *6mo)*
	*“As the woman just gave birth like that, it is hot food (TZ) they will give her but not cold food in order to help your wound heal fast” (Bagabaga 6mo* +*)*
	*“Pouring hot water in a chamber pot and add canfo and dettol and sit on the hot water so that it will help heal the wounds. If you don’t do this, it can affect you.” (Kanvili* < *6mo)*
	*“After giving birth there are wounds in you so they will give you drugs so what you will do to help yourself is to take the medications according to how it was prescribed.” (Choggu 6mo* +*)*
	*“I have never practiced any cultural practices but I heard that when a woman delivers and have not enough breast milk, they will mix millet flour with water and use it to massage the breast of the woman who delivered.” (Bagabaga* < *6mo)*
	*“They will buy fresh cow milk in Islam they will write some verse and dissolve it with the milk in a bowl for you to take it in the morning before breakfast.” (Bagabaga* < *6mo)*
	*“When the grandmothers are there, they do it because they are those practicing them. When I delivered this child, I didn’t have breast milk even today, so mixed warm water with shear butter to feed the child.” (Kalpohin 6mo* +*)*
**Inadequate Knowledge of Postpartum Danger Signs**	*“Yes, they always tell us when you go home and you are bleeding or when you are feeling dizzy or severe headache that you come back and report.” (Choggu 6mo* +*)*
	*“When your BP rises that you can come back to the facility.” (Kalpohin* < *6mo)*
	*“You can also experience headache all the time after birth.” (Kanvili* < *6mo)*
	*“If the blood is too much, they will put cotton in the wound to stop the bleeding. So, if you forget about the cotton they put in you during the surgery, it will begin to smell when it is rotten, which means you have to go to hospital.” (Bagabaga 6mo* +*)*
	*“When you go home and detected that the child is exhibiting some strange signs you need to go back to the facility.” (Bagabaga* < *6mo)*
	*“If the baby is having difficulty in breathing, you can take him to hospital.” (Choggu* < *6mo)*
**Barriers in Accessing Postpartum Care**	*“They just come to work and relax in their chairs.” (Kanvili* < *6mo)*
	*“When you come to the facility during labor at night you will call the nurses on phone or knock on their doors but you won’t get any response. Some of the nurses will be shouting at you (their utterances) toward the mothers especially during labor can prevent a pregnant woman from accessing health care services at the facility.” (Bagabaga 6mo* +*)*
	*“You can go to hospital; the nurses are many but some of them doesn’t have time for us. At times a woman may be at labor, and they call the nurses and none of them will mind her.” (Bagabaga* < *6mo)*
	*“When I met her, she didn’t mind me but was rather talking on her phone. So, when she finished, and people were talking to her about her behavior toward me, I told them to leave her because ‘every person dies but nurses never die’. So, she became annoy and threw my folders away.” (Bagabaga* < *6mo)*
	*“When you are in labour and you go to the hospital where the nurses will care for you, it is better. When I was in labour, I went to HABAANA where there was attention for me.” (Kanvili* < *6mo)*
	*“The reason why people don’t want to go hospital is that when they go to hospital they won’t give them the drugs free, unless they buy with their money. So, they prefer going to the drug store to just complain and buy with their money and go home, is better than going to the hospital. Even the health insurance you will send it and it is nothing.” (Kalpohin* < *6mo)*
	*“Truly, someone may fall sick but the means to get to the hospital is the problem. If she has no money to transport herself to the hospital, it is a problem.” (Kanvili* < *6mo)*
	*“Like today for instance, many people came and they asked them to go back and come on Monday because there is no injection forgetting that nobody knows what will happen even before Monday.” (Bagabaga 6mo* +*)*
**Experiences of Poor Mental Health**	*“If you the mother is giving birth for the first time, some babies can cry a lot. So, in the night, the cry of the baby will make you sad.” (Bagabaga 6mo* +*)*
	*“You feel very uncomfortable when the child is not feeling well. They advise that we should not give them grip water again that it is not good for the children so when that happens you the mother will cry because you don’t know how you will handle it. Mostly that is our problem.” (Kalpohin 6mo* +*)*
	*“..Meanwhile you see your colleagues’ children neatly dressed but you can’t afford for your child, you will ask yourself why God has put you in that situation, and eventually you will feel if God had taken your life, it would have been better.” (Bagabaga 6mo* +*)*
	*“You see, in life we are not equal; some are rich others are poor. So, if a poor person like me gives birth my worry is how I will be able to take care of the child until he or she becomes an adult just like the parents took care of me until I become who I am today. I think about it a lot.” (Bagabaga 6mo* +*)*
	*“You don’t get any help or support to take care of you and the baby, you can be sure of your sadness” (Bagabaga 6mo* +*)*
	*“Sometime you will wake up and there is nothing to take care of the children, and the man has not provided, so you the mother will definitely think negatively.” (Kanvili 6mo* +*)*
	*“Even if the child annoys you, you carried the pregnancy and knows how it feels not to talk of the day of delivery. There is no day that you will hate the child even when you beat the child for annoying you, you always remember the pain you go through during the pregnancy and the delivery.” (Bagabaga* < *6mo)*
	*“If you deliver through surgery, you cannot forget it.” “Sometimes you don’t expect someone to do something to you, but when it happens you remember it.” (Kanvili* < *6mo)*
	*“What I consider to be sad is when you deliver and God takes back the baby, then you will be sad by all means, otherwise I don’t see anything sad about giving birth.” (Choggu* < *6mo)*
	*Group Facilitator: Do moms feel like they are really having a tough time, so much so that they may not want to be here or keep going?* *Participants: (All in unison) No!* *“As for ending your life, NO. We pray to God to protect you against that one.” (Kalpohin* < *6mo)*
	*No. I can’t think of killing myself.” (Bagabaga* < *6mo)*
**Postpartum Educational Needs**	*“I will want us to talk about good health of our babies, we the mothers, the food we should be eating to be healthy, personal hygiene, and how to space our births.” (Bagabaga 6mo* +*)*
	*“Someone may think about how to control her birth, give birth to a reasonable number of children and take good care of them and yourself.” (Choggu* < *6mo)*
	*“We need education about BP to help us know our BP level. The nurses should tell us what we should be eating.” (Kanvili 6mo* +*)*
	*“Already, after birth when we come to the hospital, they tell us the kind of food we should eat to be strong for the baby to sack and look healthy, and how to ensure neatness so that you won’t give any sickness to the baby.” (Choggu* < *6mo)*
	*“What they think is good for mothers, especially the kind of food mothers should eat to be strong.” (Choggu* < *6mo)*
	*“When you discharged and there is no breast milk, you can prepare tea with a lot of milk and take or you prepare porridge with millet flour and take it and you will have enough breast milk.” (Kalpohin 6mo* +*)*

### Theme 1: Baby-focused postpartum care

There appeared to be a misunderstanding or lack of knowledge of what postpartum care means. Most women understood postpartum care to mean only baby care and raising the child. Women did not seem to know that postpartum care should include the care of the mother. Below are examples of what women said when they were asked to discuss what postpartum care entails:

“What postpartum care entails is that you may want to take good care of the baby and you are not well to do because most of us are unemployed, so you can’t do it. We are supposed to take good care of them. On the part of breast feeding, we should keep our breasts neat. (Participant 4, Kalpohin <6mo)”

“Making sure the baby is healthy.” (Participant 5, Choggu < 6mo)

Postpartum care was basically non-existent for the mother. However, postnatal care was provided to the baby when women attended their postnatal care visits. Many women described their postpartum care experience as baby immunizations and weighing of the baby.

*“Is there anything after postpartum? There is nothing after postpartum unless you always go for weighing and they educate you on how to care for your baby and yourself.” (Participant 4, Choggu 6mo*+*)*

Aside from weighing and baby immunizations, many women’s experiences with postpartum care also included baby-focused education on breastfeeding, baby nutrition, hygiene, weighing, baby care, and family planning.

*“When I go they encourage me as to how to breastfeed the child, how to seat and breastfeed the child, the kind of food I should prepare for the child to nourish, and myself the kind of food I should eat so that there will be enough breast milk for the child.” (Participant 5, Choggu, 6mo*+*)*

*Some p*articipants disclosed that there is generally no care for the mother during postnatal care visits unless the mother specifically seeks care for herself. Even when mothers request care for themselves during postnatal care visits, they are told to go to the outpatient department (OPD). In responding to whether they have received care for themselves since delivery, one woman said:

“No, they don’t check on our health.” (Participant 2, Choggu, 6mo +)

### Theme 2: Postpartum practices

Participants discussed various practices at home. These included personal hygiene, keeping their homes clean, sitting on hot water, taking prescribed medications, abdominal massage, and eating hot foods. Most women described postpartum practices related to healing and recovery, and breastmilk-related practices.

For postpartum healing and recovery, participants discussed taking medications, eating hot foods, sitting on hot water to expel blood from the womb, laceration care, and personal hygiene. Most women believe that there is a wound in the uterus after giving birth, and certain practices can help to heal the wound for recovery. These practices include eating hot foods, drinking hot liquids, sitting on hot water (often with substances added to the water), and massaging the uterus with hot water. The following are some quotes from participants on healing and recovery.

*“Take your medications and squat on the hot water to regain your health.” (Participant 3, Bagabaga* <*6mo)*

*“Massaging your stomach or abdomen with hot water will help the wounds to heal fast.” (Participant 4, Bagabaga 6mo*+*)*

*“As the woman just gave birth like that, it is hot food (TZ) they will give her but not cold food in order to help your wound heal fast.” (Participant 1, Bagabaga, 6mo*+*)*

There were various practices for increasing breastmilk production. These included massaging the breast with some mixture and drinking certain fluids or foods to produce more milk. A few women who struggled with breastmilk production fed their babies with warm water mixed with shea butter.

*“They will also give you hot ‘kanwa koko’ (sodium bicarbonate porridge) to drink so that you will produce breast milk for the baby.” (Participant 6, Kanvili* <*6mo)*

*“After I delivered, I didn’t have breast milk and even up till today I still don’t have breast milk, so they will mix warm water with shear butter and feed the child.” (Participant 7, Kalpohin* <*6mo)*

### Theme 3: Inadequate knowledge of postpartum danger signs

Overall, knowledge of postpartum danger signs among participants was low. Very few participants were able to discuss danger signs of postpartum complications. Among participants who responded to the discussion, knowledge of danger signs centered around hemorrhage, infection, and high blood pressure. Other life-threatening complications such as postpartum depression, pulmonary embolism, and venous thrombosis did not come up in the discussion among participants.The most frequently mentioned danger sign among participants was severe bleeding after delivery. In addition to bleeding, participants also talked about dizziness, headaches, and high blood pressure. In addition, some participants also mentioned danger signs to look out for in their babies. Below are some quotes from participants on danger signs postpartum.

*“If there is continuous bleeding after delivery, you can come back to the facility.” (Participant 5, Kalpohin* <*6mo)*

“If the baby has high body temperature” (Participant 6, Choggu < 6mo).

### *Theme 4:* Barriers in accessing postpartum care

Participants noted several reasons why it was difficult to seek out medical care or receive medical care once they attended a health center. There appeared to be two main types of barriers to access care: interpersonal factors and lack of resources (e.g., lack of money, insurance, transportation).

*Many women’s* negative interactions with healthcare workers often involved feeling ignored and not taken care of while at healthcare facilities. Some participants also described speaking up and asking for assistance but feeling that their requests were not taken seriously. When speaking about barriers in obtaining care for themselves, one participant said:

*“As for the challenges, they are plenty. You may come to meet a nurse with hot temper and the utterance alone will scare you, and anytime you want to come here, you are worried.” (Participant 5, Kanvili* <*6mo)*

*Some p*articipants noted sometimes interactions with healthcare workers went beyond feeling ignored or not taken seriously into more hostile interactions. Others also described corruption and nepotism as factors affecting the quality of care an individual may receive at a healthcare facility.

*“At the hospital, whom you know counts a lot. If you don’t know anybody at the hospital you will not get proper attention.” (Participant 4, Choggu* <*6mo)*

Another significant barrier to receiving care was the lack of resources, especially if participants lacked money, insurance, and transportation. Most participants tended to be interested in receiving health care but may not have had the resources to receive care. For example, many participants noted that even if they went to healthcare facilities, they were still expected to pay for medication at a drugstore, which was not always possible. It also appeared that healthcare systems were juggling resource burdens. The following are some example quotes from participants on lack of resources.

“The reason why people don’t want to go to the hospital is that when they go to hospital they won’t give them the drugs for free, unless they buy with their money. So they prefer going to the drug store to just complain and buy with their money and go home, is better than going to the hospital. Even the health insurance you will send it and it is nothing.” (Participant 3, Kalpohin <6mo)

*“There are so many people who are not well to do so access to health is very difficult because there is no money to pay. Immediately you deliver the NHIS also seizes to work unless you go and renew and that one too is a challenge.” (Participant 4, Choggu 6mo*+*)*

### Theme 5: *Experiences of poor *mental health

When asked about mental health issues, participants often described feelings of sadness, being alone and without resources, or experiencing stress due to the demands of postpartum care. Sometimes sadness or worry was normalized as part of what comes with becoming a mother. Some women also noted struggles with taking care of the child in the postpartum period and this could lead to sadness or lack of peace. Often poor mental health was strongly associated with a lack of money or support from the husband and/or family. There was also discussion of traumatic memories. Some participants noted not wanting to remember the birth experience due to pain associated with the birth experience and perinatal period. Below are a few example quotes from participants:

*“For instance, when I deliver I don’t have enough breast milk for one week. He/she will be crying and you don’t want to give him/her anything else. So you will see him/her growing lean. So this alone is a problem and you will regret giving birth and the peace that you had leaves you.” (Participant 6, Choggu 6mo*+*)*

*“Life can become hard for you especially if your husband doesn’t give you money, and where you are your parents are not there, and your in-laws too don’t have. Meanwhile you see your colleagues’ children neatly dressed but you can’t afford for your child, you will ask yourself why God has put you in that situation, and eventually you will feel if God had taken your life, it would have been better” (Participant 1, Bagabaga 6mo*+*)*

*“If you deliver through surgery, you cannot forget it.” (Participant 7, Kanvili_below 6 months)*When discussing mental health, there was often a minimizing of negative feelings or focusing on religion as a way to cope with stress rather than allowing negative emotions to be present.

*“Some are wishing for what you have but God has not given them. So, if you have a child and there is no money, just manage with your torn cloth and be thankful to God.” (Participant 7, Bagabaga 6mo*+*)*

When asked about ending their lives due to overwhelming negative thoughts/emotions, most strongly denied thoughts or allowing thoughts to persist. However, a few participants endorsed that when things get tough, thoughts of not being here or ending their life are sometimes entertained:

*“We Dagomba women, the man will wake up and go out without asking about what the children will eat. You the mother will know how you will do and feed the children. So, when you don’t have [money]to feed the children, you will begin to think that way. Because this child will come with his problem and another child also comes with a problem, so you finally think the unthinkable since you cannot do what they want.” (Participant 7, Kanvili 6mo*+*)*

In summary, women endorse struggling with feelings of sadness, loneliness, and desperation, especially when lacking support or resources. There is a tendency to focus on the positive and utilize religious practices to cope with these feelings. There is an acknowledgment by some on how severe these thoughts can get with regard to suicidality but mostly participants deny engaging in suicidal ideation or passive thoughts of death.

### Theme 6: Need for postpartum education

Overall, participants reported that their educational needs centered around best practices in caring for their children, family planning, and exchanging ideas. Mostparticipants knew some of this information already and wanted to enrich their prior knowledge. Additionally, a focus on personal hygiene was a theme throughout discussions. There was a particularly strong focus on requesting knowledge on family planning, education on taking medicine to manage medical conditions, and eating specific food postpartum to ensure healthy children and strong mothers:Lastly, participants wanted to know about breastfeeding, especially if their breastmilk wasis not coming in and how to help the baby in those situations. Below are quotes from participants on educational needs:

*“Some of us are illiterate and we don’t know much about family planning. We don’t know how to use it to control our births, so we need education.” (Participant 7, Bagabaga 6mo*+*)*

*“At times when you are not happy you will not be able to produce enough breast milk. If you are not peaceful it will make production of breast milk difficult. So they should add that to their education.” (Participant 6, Kalpohin* <*6mo)*

*“For example, like what we have discussed and this woman said she doesn’t have enough breast milk, so if we gather you can educate us about that. We can share our experiences and also receive some education from you too.” (Participant 4, Kalpohin* <*6mo)*

## Discussion

This qualitative study utilized thematic analysis to examine eight focus groups of postpartum women in Ghana. Critically, participants had limited knowledge of postpartum danger signs, including lack of knowledge of life-threatening mental and physical complications such as postpartum depression, pulmonary embolism, and venous thrombosis.

Participants described a lack of postpartum care, with very little discussion on care for themselves outside of caretaking of the infant. Participants felt that poor interpersonal interactions interfered with obtaining postpartum care if needed and that mental health needs, if recognized, were not met. In regard to ongoing education, participants were most interested in best practices in surrounding caring for children, family planning, and exchanging ideas.

There was a lack of knowledge on danger signs for common causes of morbidity and mortality in the postpartum period. This may be due to the lack of education on several important topics provided in too few postpartum visits. The first three months postpartum (often considered the “fourth trimester”) are a period of substantial change and often new health problems for women [20, 21]. During these visits, women receive insufficient information on birth control post-delivery, emotional, and mental health issues, sexual matters, and other postpartum challenges [20, 22]. A recent study shows that perinatal women prefer an increase in postpartum visits and between-visit contact [23]. For example, in the United States, there is often one recommended postpartum visit six weeks after childbirth, of which less than half of women attend and is not regularly reimbursed by insurance.Women (and their families) need a strong support system post-delivery that combines different domains of medicine, social and family support, and mental health services [20, 24]. For example, Tully et al. (2017) proposed a postpartum care model that includes the domains of medications, substances, and exposures; mood and emotional well-being; infant care and feeding; sexuality, contraception, and birth spacing; sleep and fatigue; physical recovery from childbirth [21]. This study adds to the growing number of studies describing the knowledge gap and cultural practices that do not focus on the mother in the postpartum period and how improved models of care are necessary.

The biggest barriers to obtaining adequate postpartum care were primarily interpersonal.s. Participants across focus groups noted several examples of staff saying hurtful statements to them during visits or not being attentive. This highlighted the importance of the relationship with healthcare workers and how it can dramatically affect a woman’s comfort in seeking out care as needed. Other studies of perinatal women in SSA countries have also highlighted negative treatment from healthcare workers as a deterrent to remaining in care during the postpartum period [25]. Future research may need to incorporate interventions that improve communication between healthcare workers and postpartum women to have a stronger, more open relationship and improve service delivery.

One of the most striking findings was how when asked about care in the postpartum period, participants understood postpartum care as caring for the baby only, not caring for themselves. Less than ideal postpartum care, often with a focus on the baby and lack of attention on the mother from providers or the healthcare system, is a common theme in the postpartum period across the world, including in SSA countries [26, 27]. In our study, even when mothers request care for themselves during postnatal care visits, they are not receiving it. This is consistent with other studies of postpartum women reporting that they are interested in face-to-face care sooner in the postpartum period to address maternal health issues before they can become problematic at 6 weeks postpartum [28]. Future research should investigate how to match clinical practice with postpartum women’s desire for more care earlier in the postpartum period.

This lack of care extended to mental health. Participants in focus groups endorsed mental health concerns indicative of possible postpartum depression (PPD), anxiety, and traumatic stress that are very common in the postpartum period, including in Ghana and other SSA countries [23, 29, 30]. However, a common theme when discussing mental health was that participants minimized mental health concerns and instead noted that postpartum women should focus on religion when feeling negative emotions or just feel happy and/or grateful for having a child. Discussion of severity and experience of mental health may be stigmatized. This was highlighted when discussing suicidality in the focus groups; women forcefully rejected discussion of suicidal thoughts, even though thoughts of suicidality among postpartum women who experience depressive symptoms are not uncommon [31].

Stigma around acknowledging or discussing mental health may minimize the importance of seeking care for postpartum mental health. This was seen in a recent cross-sectional survey that assessed postpartum depressive symptoms in over three thousand postpartum women in rural Ethiopia [29]. For women with high PPD symptoms, less than 5% had obtained mental health care and only about 13% had been in contact with any health service. Postpartum women in Ghana are experiencing common mental health symptoms, but there may be a reluctance to acknowledge them, which may impair getting help if needed. Future research should explore how to increase awareness about postpartum mental health issues and how to receive care.

### Limitations

This study was limited by the small sample size and study context. Additionally, focus group sessions can have interpersonal dynamics that prevent some participants from openly sharing relevant views, especially when there are differences in age or parity among group members (e.g. nulliparous vs. multiparous, younger mothers vs. more experienced mothers). Future research should include examination of focus group dynamics alongside content to reduce this limitation.

In conclusion, this study demonstrated the importance of understanding the postpartum needs of women and the challenges to obtaining quality postpartum care to design better, more effective interventions. Thus, we recommend that further research is conducted in other settings to determine if the themes generated are consistent or different across various settings. It will also be useful to obtain and compare the perspectives of maternity care providers.

## Data Availability

The datasets used and/or analyzed during the current study are available from the corresponding author on reasonable request.

## Abbreviations

MMR: Maternal Mortality Ratio
SSA: Sub-Saharan Africa
ANC: Antenatal Care
PNC: Postnatal Care
WHO: World Health Organization
PPD: Postpartum Depression
